# Neuroprotection by Polynitrogen Manganese Complexes: Regulation of Reactive Oxygen Species-Related Pathways

**DOI:** 10.1038/srep20853

**Published:** 2016-02-09

**Authors:** Chunxia Chen, Jing Cao, Xiaoyan Ma, Xiaobo Wang, Qiuyun Chen, Shihai Yan, Ningwei Zhao, Zhirong Geng, Zhilin Wang

**Affiliations:** 1 State Key Laboratory of Coordination Chemistry, School of Chemistry and Chemical Engineering, Collaborative Innovation Center of Advanced Microstructures, Nanjing University, Nanjing 210093, P.R. China; 2 School of Chemistry and Chemical Engineering, Jiangsu University, Zhenjiang 212013, P.R. China; 3 Department of Pharmacology, Jiangsu Province Hospital of Chinese Medicine, Nanjing 210029, P.R. China; 4 Biomedical Research Laboratory, Shimadzu (China) Co., Ltd, Shanghai 200052, P.R. China

## Abstract

Cell death in the central nervous system causes neurologic diseases, in which reactive oxygen species (ROS) play a critical role by either inducing cellular oxidative stress or by increasing the cell tolerance against insult. Neurologic diseases may potentially be treated by regulating ROS levels in a certain range with small molecules. We studied preconditioning with two polynitrogen manganese complexes (**1** and **2**) to regulate intracellular ROS levels in the protection of both the differentiated rat pheochromocytoma cell line (PC12 cells) and neurons against H_2_O_2_-induced apoptosis. Pre-treatment with the two complexes attenuated the cell apoptosis caused by H_2_O_2_. And the ROS-related neuroprotective mechanisms were explored. Both complexes activate the hypoxia inducible factor-related pathways and increase the cell adaptation to oxidative stress. Pre-treatment with complex **1** eliminated intracellular ROS, which also activated antioxidase system, while short-term incubation of complex **2**, generated low levels of ROS leading to cell survival.

Neurologic diseases, including stroke, Alzheimer disease, Parkinson disease and ischemic stroke, are leading causes of death and long-term disability worldwide^1^. These diseases are caused by neuronal injury due to excitotoxic cascade, free radical damage, inflammation and delayed neuronal death^2^. Among these molecular mechanisms of neuronal cell death, reactive oxygen species (ROS), as free radicals, have been implicated to play critical roles in the pathophysiology of neurologic conditions^3^. On the one hand, ROS, which produced mainly within the mitochondria electron transport chain, lead to ischemic cell death-involved brain injury by destructing cellular proteins, lipids and DNA thereby disrupting normal cellular signaling and gene regulation^4^,^5^. ROS generation is able to activate signaling cascades mediating physical and chemical stresses in cells^4^. Hence, inhibiting the formation of highly reactive species can limit the damages to cellular components. Enhanced expressions of free radical scavengers, such as catalase or superoxide dismutase (SOD), exert neuroprotective effects by protecting neurons from oxidative damage and by promoting neuronal survival^6^,^7^. On the other hand, ROS are responsible for the stabilization of a transcription factor called hypoxia inducible factor (HIF)^8^,^9^, which is a master regulator of oxygen homeostasis^10^. Involved in the activation of HIF, ischemic/hypoxic preconditioning with mild, non-damaging stress induces tolerance against a subsequent severe insult, which is beneficial to protect the brain from oxidative damage^11^. Therefore, low dose of exogenous H_2_O_2_ triggering HIF-1α expression contributes to neuroprotection, which partly mimics the ischemic or hypoxic preconditioning^12^, indicating that not all consequences of ROS insult are deleterious. Known therapeutic options or strategies against neuronal injury are very limited, without considering the dual roles of ROS. Therefore, the aim of this study was to manipulate ROS production by pharmacological interventions, and to explore the ROS-HIF related neuroprotection mechanism, which will help to discover novel drugs for neurologic diseases therapy.

Manganese (Mn), containing several valence states (such as Mn^II^, Mn^III^, Mn^IV^, Mn^V^, Mn^VII^), is a co-factor required for many redox enzymes^13^,^14^. Polynitrogen Mn complexes have been found as active sites in a number of metalloenzymes, such as Mn catalase or Mn-SOD^13^,^15^, because Mn ions can be both oxidized and reduced by ROS^16^. Previous studies have shown that synthesized MnSOD/catalase mimics were protective in the model of cerebral ischemia^17^ and mesencephalic neuronal-glial cultures^18^, increasing the cell viability by reducing the intracellular ROS^19^. Given the ability of reacting with ROS, we studied the neuroprotective effects of two structural distinct polynitrogen Mn complexes **1** and **2** (structure formulas shown in Fig. 1). Complex **1**, as a dinuclear manganese (Mn^III^Mn^IV^) complex with the [Mn_2_(μ-O)_2_μ-OAc] core, mimics the highly catalytically catalase in efficiently scavenging H_2_O_2_^15^,^20^. Complex **2**, which is a Mn^II^ complex containing N-substituted di(picolyl)amine ligand, can also dispose H_2_O_2_ through being oxidized^21^. Instead, studies also found that Mn^II^ complexes that sharing the same ligand with **2** can stimulate the generation of ROS in cells by interacting with the mitochondria^22^,^23^. Thus the balances between up- and down- regulation of endogenous ROS levels caused by complex **1** and **2**, which are responsible for neuronal cells survival, are worth exploring.

In the present study, a differentiated rat pheochromocytoma cell line (PC12 cells) and rat hippocampal neurons were both used as *in vitro* neuron model. H_2_O_2_ was used to induce oxidative injury in the cells. We investigated preconditioning of the two complexes in protection of neuronal cells against H_2_O_2_-induced cell death, and demonstrated that the complexes attenuated cell apoptosis at lower doses than reported drugs did. Since the neuroprotective pathways of many potentially eligible compounds have barely been clarified, we also explored the ROS-related signaling pathways to shed light on the neuroprotective mechanism of the two complexes. We have not only found out that both Mn complex **1** and **2** activated HIF and its downstream genes thereby increasing the cell tolerance to H_2_O_2_, but also presented a dialectical view of ROS regulation on neuroprotection. Our study provides a new train of thought in discovering novel drugs for neurologic diseases therapies.

## Results

### Cytotoxicity of Mn complexes 1 and 2

Initial experiments were performed to determine the cytotoxicity profiles of Mn complexes for differentiated PC12 cells (See Supplementary Fig. S1). The two complexes had different effects on PC12 cells viability. Complex **1** at 0–200 μM barely affected cell survival, and longer incubation of **1** at below 150 μM even promoted cell survival. The IC_50_ values of **1** were about 600 and 500 μM at 12 and 24 h, respectively. But unlike **1**, complex **2** induced evident PC12 cells death. Only 30% of the cells survived during 12 h treatment of **2** at 200 μM. In addition, the cells were more prone to death with long-term treatment. The IC_50_ values of **2** corresponding to 12 and 24 h of incubation were about 20 and 15 μM, respectively. Thus, non-toxic concentration ranges of the complexes (**1**, 0.01–200 μM; **2**, 0.001–5 μM) were selected for PC12 cells and neuronal cells related treatments.

### Effects of different concentrations of H_2_O_2_ on apoptosis of PC12 cells

To explore the critical role of ROS levels in cell survival, oxidative treatment was performed by incubating differentiated PC12 cells with different doses of H_2_O_2_ for 12 h (See Supplementary Fig. S2). H_2_O_2_ played dual roles on the PC12 cell viability. Dose-dependent cell death occurred with the amount of H_2_O_2_ exceeding 100 μM, though differentiation for longer time increased cell viability against H_2_O_2_ induced apoptosis. Interestingly, however, using H_2_O_2_ at lower doses (3 to 50 μM) did not reduce cell survival. Moreover, when the differentiation time of PC12 cells was longer, 50 μM H_2_O_2_ even increased cell viability to 99%, which exceeded 10 or 20 μM H_2_O_2_ did. The results confirmed that low-dose H_2_O_2_ showed protective effects on PC12 cell survival while higher concentrations of that induced considerable cell death. As a result, 100–200 μM H_2_O_2_ treatment was chosen for the model of oxidative injury as it decreased the viability of PC12 cells by 40–60% compared with that of control.

### Protection of preconditioning with Mn complexes against H_2_O_2_-induced death of neuronal cells

**Figure 2:**
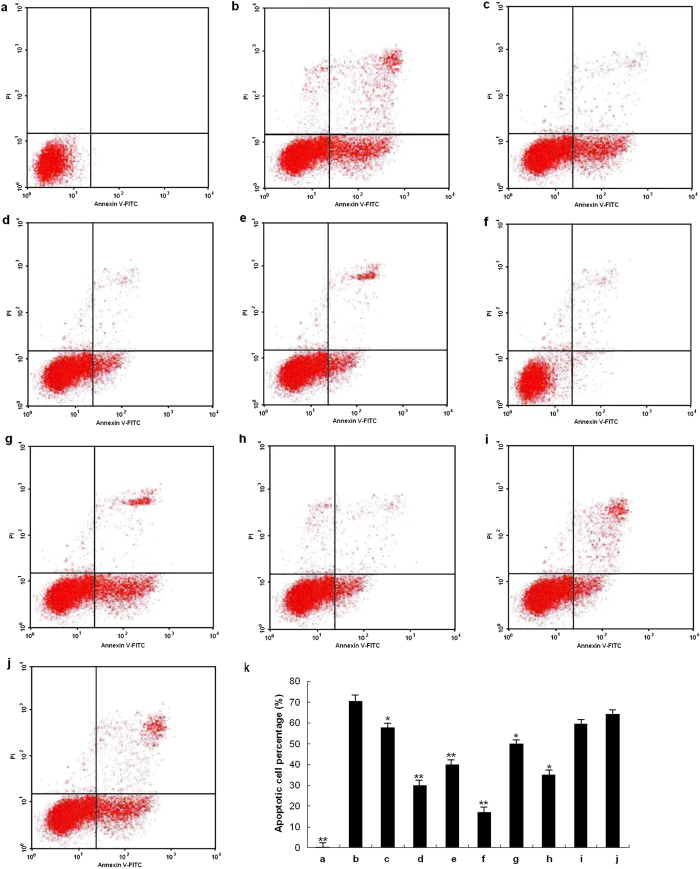
Mn complexes 1 and 2 protected neurons against H_2_O_2_-induced cell apoptosis. Cell apoptosis was detected by a flow cytometer. The neurons were pre-incubated with (**a**) neuronal medium (control), (**b**) neuronal medium, (**c**) **1** (1 μM) for 12 h (**d**) **1** (10 μM) for 12 h, (**e**) **1** (1 μM) for 24 h, (**f**) **1** (10 μM) for 24 h, (**g**) **2** (0.1 μM) for 12 h, (**h**) **2** (1 μM) for 12 h, (**i**) **2** (0.1 μM) for 24 h and (**j**) **2** (1 μM) for 24 h, and then treated with H_2_O_2_ (200 μM) for 12 h (**b–j**) Statistical analysis of the apoptotic cell percentage. The data are presented as mean ± SD of three independent experiments. (Student’s T. Test, *P < 0.05, **P < 0.01 vs. group (**b**)).

To determine the protective effects of the two Mn complexes against cell oxidative injury, both PC12 cells and neurons were treated with them in the above-mentioned concentration range for 12 or 24 h, and subsequently exposed to 200 μM H_2_O_2_ for 12 h. The cell survival was measured by both MTT assay and Annexin V/PI double staining. 200 μM H_2_O_2_ was chosen to induce a more evident neuron apoptosis effect. As shown in Fig. 2, only about 30% of neurons survived upon H_2_O_2_-induced oxidative damage. **1** increased cell viability to 40–70% for 12 h precondition and to almost 90% for 24 h precondition. In contrast, pre-treatment with complex **2** for 12 h rescued the cell survival rate to over 60%, but longer pre-incubation of it induced cell apoptosis. The two complexes exerted time-dependent neuroprotective effects. Preconditioning with complex **2** for 12 h was more effective than that for 24 h, while longer preconditioning time was required for **1** to be more protective. Similar results were also obtained from the MTT assay on PC12 cells (See Supplementary Fig. S3). Particularly, the minimum protective doses of **1** and **2** against oxidative stress in PC12 cells were 0.1 and 0.001 μM respectively, which were lower than those of some reported Mn SOD/catalase mimics (over 1 μM, or even over 10 μM)^24^ or natural antioxidants such as salidroside (more than 8 μM), and calycopterin (25 μM)^25^,^26^. More importantly, complex **1** was less cytotoxic, as evidenced by the protective effects of both 12 h and 24 h preconditioning on neuronal cells against oxidative death. Double stained PC12 cells were analyzed by fluorescence microscope (See Supplementary Fig. S4). In addition, both neurons and PC12 cells contained the same neuronal marker (See Supplementary Fig. S5), indicating that the PC12 cells can be used as a model of neuronal cells *in vitro*.

The expression of Bcl-2 has been correlated with the initiation of a cascade which inactivates caspases such as Caspase-3^27^. Most cellular apoptosis pathways converge on Caspase-3 activation^28^. In this study, pre-conditioning with Mn complexes **1** and **2** was potentially neuroprotective. Differentiated PC12 cells were treated with drugs and H_2_O_2_ for certain time, and the whole cell protein was collected for western blot analysis to study the mechanism underlying the neuroprotective effect. As shown in Fig. S6, the expression levels of Bcl-2 of the H_2_O_2_ treatment group were remarkably reduced compared to those of the control group, whereas 12 h pre-treatment of **2** and both 12 h and 24 h pre-treatment of **1** dramatically increased the protein levels of Bcl-2. Moreover, in the cells pre-treated with **1** for 24 h and **2** for 12 h, the levels of uncleaved Pro-caspase-3 were increased dose dependently, compared to that in only H_2_O_2_ treated cells. It was worth mentioning that 24 h dosing of **2** caused cleavage of Caspase-3 dose dependently, as verified by the decrease of Pro-caspase-3 protein levels, which was in agreement with the time-dependent protective effects of **2**.

### Time course analysis of intracellular ROS levels changes

Both **1** and **2** could protect neuronal cells against H_2_O_2_-induced injury at certain doses but with different pre-treatment time. According to our study, low doses of H_2_O_2_ were beneficial for neuronal cells’ survival. Therefore, time course analysis of the ROS levels affected by the two complexes was investigated. The neurons were treated with lower and higher doses of catalase (0.01 and 0.1 mg/ml), complex **1** (1 and 10 μM) and **2** (0.1 and 1 μM) for 1 h, 12 h and 24 h, respectively, and collected for incubation with DCFH-DA at 37 °C^29^,^30^. Treatment only with normal culture medium containing neither catalase nor complexes served as control. Flow cytometer was then used to analyze the ROS levels changes affected by catalase and its model complexes.

**Figure 3:**
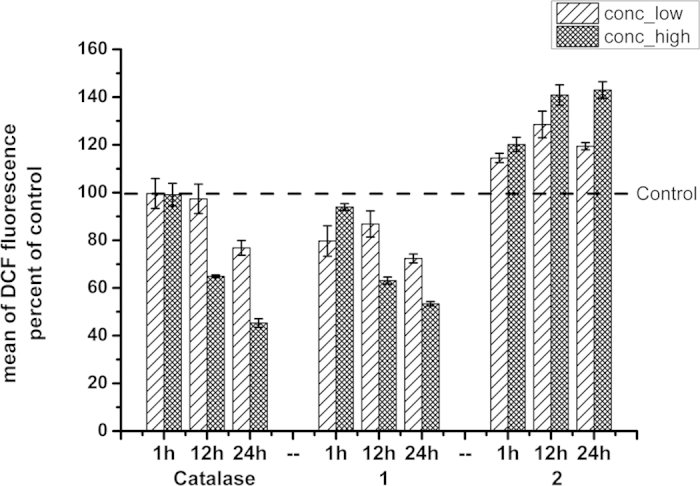
Time course analysis of intracellular ROS levels changes caused by catalase and Mn complexes **1** and **2** treatment. The neurons were incubated with two concentrations of catalase (CAT), complex **1** and complex **2** for 1 h, 12 h and 24 h, respectively. Collected cells were incubated with DCFH for 20 min at 37 °C. The fluorescence of DCF was measured by a flow cytometer. ROS levels are expressed as a histogram of the DCF fluorescence generated by 20,000 cells. Treatment with only normal culture medium without drug stimulation served as control. The fluorescence intensity of each sample was expressed as the percent (%) of control values, which were shown in dotted line at the value of 100%. The columns representing lower doses (0.01 mg/ml of CAT, 1 μM of **1** and 0.1 μM of **2**) treatment were filled with the sparse slashes pattern, while that representing higher doses (0. 1 mg/ml of CAT, 10 μM of **1** and 1 μM of **2**) treatment were filled with the dense pattern. The data are presented as mean ± SD of three independent experiments (Student’s T. Test, *P < 0.05, **P < 0.01 vs. control).

As shown in Fig. 3, different incubation time of **1** and **2** had distinct influence on intracellular ROS levels. 1 h incubation of complex **1** did not affect ROS levels significantly. Prolonged incubation of **1**, however, induced dramatically decrease of intracellular ROS just like the catalase did. 24 h treatment of **1** (10 μM) evidently decreased the ROS levels by 40%, and the catalase (0.1 mg/ml) induced even more significant ROS scavenging effects. On the contrary, incubation of complex **2** for 1 h, 12 h and 24 h, at both 0.1 μM and 1 μM stimulated ROS generation in neurons by 10–40%, which was quite different with the ROS scavenging drugs, suggesting that the intracellular ROS would sustain in higher levels after long-term treatment of **2**. Probably, longer pre-incubation of **2** might give rise to cytotoxicity by producing excessive ROS, which also activate some signaling pathways that related to cellular oxidative stress.

### Effects of Mn complexes on the mRNA levels of HIF-1α and HIF target genes in cultured cells

**Figure 4:**
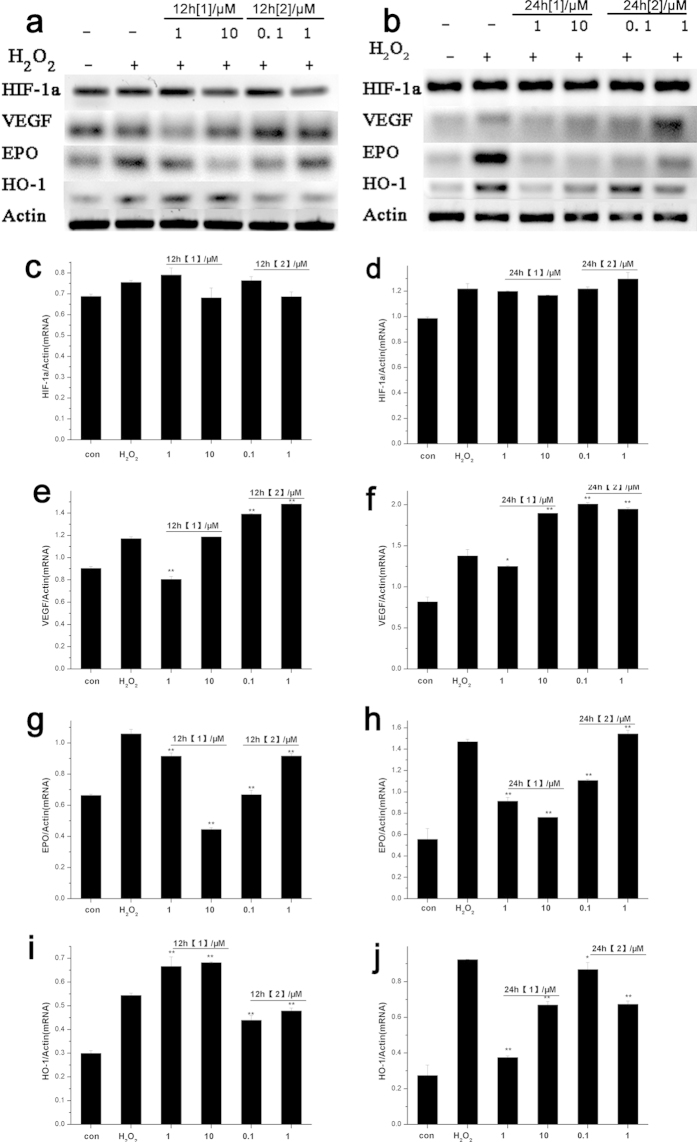
Effects of Mn complexes pre-incubation on mRNA levels of HIF-1α and HIF target genes in neuronal cells. The differentiated PC12 cells were pre-conditioned with Mn complexs **1** and **2** for 12 h (**a**) or 24 h (**b**), and treated with H_2_O_2_ (100 μM) for 12 h. HIF-1α and HIF target genes expression were detected by reverse transcription-PCR. (**c**,**d**) Statistical analysis of HIF-1α gene expression levels. (**e**,**f**) Statistical analysis of VEGF gene expression levels. (**g**,**h**) Statistical analysis of EPO gene expression levels. (**i**,**j**) Statistical analysis of VEGF gene expression levels. The expressions of mRNA levelss were given as HIF-1α/β-Actin, VEGF/β-Actin, EPO/β-Actin and HO-1/β-Actin ratio. The data are presented as mean ± SD of three independent experiments (One Way ANOVA, *P < 0.05, **P < 0.01 vs. only H_2_O_2_ stimulation but without any pretreatment).

HIF-1α, whose expression is triggered by exogenous H_2_O_2_, was studied because it is neuroprotective by inducing cell tolerance against oxidative damage^27^. To understand the neuroprotective signaling pathways, reverse transcription-PCR was employed to analyze the mRNA levels of HIF-1α and several known HIF-1α target genes (VEGF, EPO, and HO-1) in the cells exposed to the two complexes (Fig. 4). The PC12 cells were pre-treated with **1** and **2** for 12 h or 24 h and exposed to 100 μM H_2_O_2_ insult for 12 h. The results demonstrated that the HIF-1α mRNA levels were almost unchanged no matter 12 h or 24 h pre-conditioning of Mn complexes in neuronal cells. H_2_O_2_ induced the accumulation of HIF downstream genes VEGF, EPO and HO-1 mRNA compared with control group. 12 h pre-conditioning of complex **1** elevated VEGF and HO-1 mRNA expression at dose dependently, but the mRNA levels of EPO could only sustain with lower dose of **1**. Time-dependent up-regulation of mRNA levels only occurred on VEGF for pre-treatment of both **1** and **2**. Interestingly, 24 h pre-conditioning of complex **2** up-regulated EPO and HO-1 mRNA levels more significantly. The results also showed that pre-incubation time for **1** and **2** induced distinct mRNA levels of HIF downstream genes.

### Effects of Mn complexes on the protein levels of HIF-1α and HIF target genes in cultured cells

**Figure 5:**
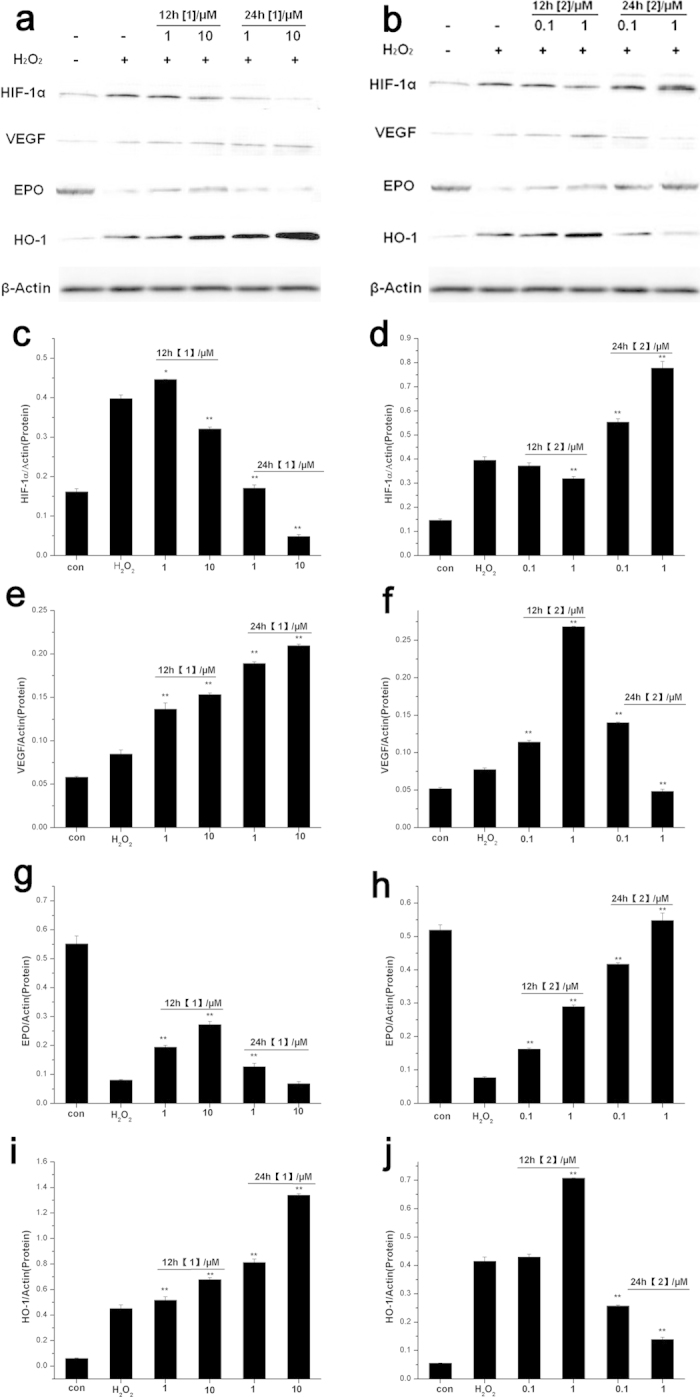
Effects of Mn complexes pre-incubation on protein levels of HIF-1α and HIF target genes in neuronal cells. The neurons were pre-conditioned with Mn complexs **1** (**a**) and **2** (**b**) for 12 h or 24 h, and treated with H_2_O_2_ (100 μM) for 12 h. The protein expression of HIF-1α and downstream genes were detected by Western-blot. (**c**,**d**) Statistical analysis of HIF-1α gene expression levels. (**e**,**f**) Statistical analysis of VEGF gene expression levels. (**g**,**h**) Statistical analysis of EPO gene expression levels. (**i**,**j**) Statistical analysis of VEGF gene expression levels. The expressions of protein levels were given as HIF-1α/β-Actin, VEGF/β-Actin, EPO/β-Actin and HO-1/β-Actin ratio. The data are presented as mean ± SD of three independent experiments (One Way ANOVA, *P < 0.05, **P < 0.01 vs. only H_2_O_2_ stimulation but without any pretreatment).

Simultaneously, we explored HIF-related signaling pathway by examining the protein levels of HIF-1α and HIF target genes: VEGF, EPO, and HO-1(Fig. 5 and Fig. S7). The protein levels of HIF-1α in Mn complexes-treated cells extracts were detected. As shown in Fig. 5, the neurons were pre-treated with **1** and **2** for 12 h or 24 h and exposed to 100 μM H_2_O_2_ insult for 12 h. H_2_O_2_ treatment elevated HIF-1α protein levels in neuronal cells. Pre-treatment with **1** at lower doses for 12 h sustained the protein levels of HIF-1α, while 24 h of preconditioning alleviated H_2_O_2_-induced up-regulation in a concentration-dependent manner. On the contrary, preconditioning of **2** induced HIF-1α protein to accumulate after H_2_O_2_ exposure. Moreover, preconditioning of **2** (1 μM) for 24 h induced significantly up-regulation of HIF-1α protein levels. Similar results on PC12 cells were shown in Fig. S7 (See supplementary information), in which complex **1** induced the up-regulation of HIF-1α protein only for short-term pre-incubation while 2 up-regulated HIF protein expressions for both 12 h and 24 h pre-treatment. Higher pre-incubation concentration (more than 1 μM) of **2** decreased HIF-1α protein expressions, implying that **2** was cytotoxic when used for long-term pre-treatment.

VEGF is one of the HIF downstream targets that having neuroprotective roles^31^,^32^. H_2_O_2_ induced VEGF protein to accumulate by 20–30% compared with the control group (Fig. 5e,f). Both 12 h and 24 h pre-conditioning of complex **1** induced up-regulation of neuron VEGF protein expression dose-dependently. Pretreatment of **2** (1 μM) for 12 h elevated VEGF protein levels significantly, but the protein levels of VEGF plummeted after 24 h pre-conditioning. The released of VEGF protein in PC12 cells supernatants were measured using an ELISA kit, which was almost in accordance with the changes in Western blot (See Supplementary Fig. S8). In general, the preconditioning time that stimulated VEGF protein release was consistent with the neuroprotective effects of the two complexes.

Besides VEGF, a number of HIF target genes, such as erythropoietin (EPO) and heme oxygenase-1 (HO-1), may also contribute to neuroprotection. EPO is a prototypic HIF responsive gene, with its multimodal neuroprotective role including anti-apoptotic, neurotrophic, angiogenic effects^33^,^34^. H_2_O_2_ negatively regulated EPO protein expression, suggesting attenuated neurotrophic effects (Fig. 5g,h). The up-regulation of EPO protein levels induced by complex **1** and **2** eliminated the neurotoxic effects caused by H_2_O_2_ exposure.

HO-1, an enzyme degrading heme to carbon monoxide, free iron and biliverdin, participates in the cell defense against oxidative stress^35^. As shown in Fig. 5i, pre-incubation of **1** significantly stimulated HO-1 protein expression dose-dependently. Especially, long-term pre-incubation of **1** (10 μM) increased the HO-1 protein levels by 150%, exhibiting notable antioxidant effects. 12 h preconditioning of **2** also induced the protein expression of HO-1. However, 24 h preconditioning of **2** decreased HO-1 protein levels, which was in consistent with the HIF-1α protein expression regulation.

### HIF-1α knockdown induced apoptotic cell death under preconditioning with Mn complexes of neuronal cells

**Figure 6:**
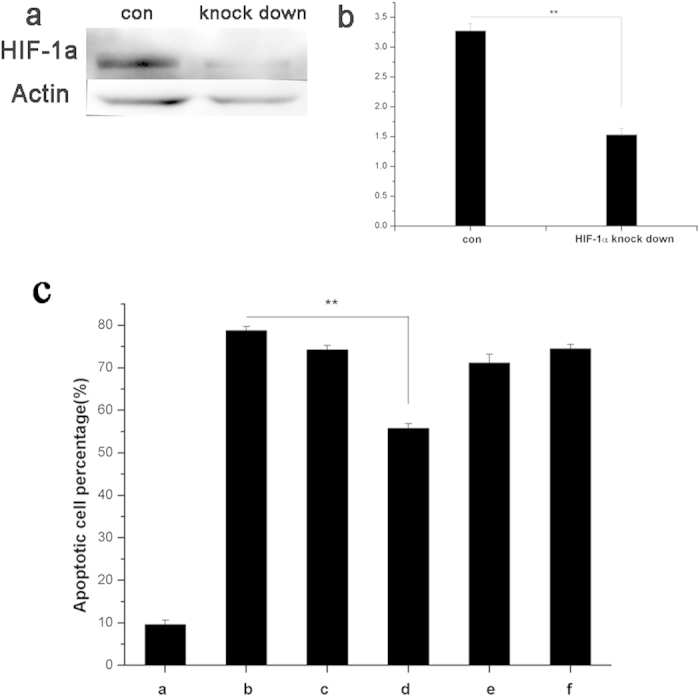
HIF-1α knockdown induced apoptotic cell death under preconditioning with Mn complexes in neuronal cells. **(a)** PC12 cells were transfected with plasmid DNA which knocked down HIF-1α protein expression and the results was tested by western blot. **(b)** Statistical analysis of the percentage of HIF-1α protein expression. **(c)** Cell apoptosis detected by a flow cytometer. After 6 h transfection, the cells were pre-incubated with (**a**) neuronal medium (control), (**b**) neuronal medium, (**c**) **1** (10 μM) for 12 h (**d**) **1** (10 μM) for 24 h, (**e**) **2** (1 μM) for 12 h and (f) **2** (1 μM) for 24 h and then treated with H_2_O_2_ (200 μM) for 12 h (**c–f**) Statistical analysis of the apoptotic cell percentage. The data are presented as mean ± SD of three independent experiments (Student’s T. Test, *P < 0.05, **P < 0.01 vs. only H_2_O_2_ stimulation but without any pretreatment).

To demonstrate the protective effects of HIF-1α in the two Mn complexes against cell oxidative injury, the PC12 cells was transfection with plasmid DNA to knock down HIF-1α protein expression. Western-blot analysis showed that the HIF-1α protein expression was about 50% knocked down in the PC12 cells after 6 hours of transfection compared with control group (Fig. 6a,b). The cell viability was estimated by Flow cytometry after 6 hours of transfection and subsequently pre-conditioning of complexes and H_2_O_2_ exposure (Fig. 6c, Supplementary Fig. S9). About 20% of neuronal cells survived upon H_2_O_2_ -induced oxidative damage. Pre-conditioning of **1** and **2** for 12 h after transfection did not increase cell viability. However, preconditioning of complex **1** for 24 h rescued the cell survival rate by 50% compared with the H_2_O_2_ group. Longer pre-incubation time of complex **2** induced more cell apoptosis compare with H_2_O_2_ group. The results demonstrated that short-term neuroprotection effects of **1** and **2** were related to HIF-related pathway while long-term neuroprotection effects of **1** did not.

## Discussion

Two polynitrogen Mn complexes had different effects by pre-treating for different hours, due to their distinct Mn valence state causing different regulations on intracellular ROS levels. Complex **1** is a dinuclear Mn(III)Mn(IV) complex with the [Mn_2_ (μ-O)_2_μ-OAc] core. As studied previously, complex **1** modeled the successive electron-transfer reaction from Mn^III^_2_ to Mn^III^Mn^IV^, and then to Mn^IV^_2_ oxidation levels, indicating that it was operative during H_2_O_2_-driving redox levels change cycles^16^. Therefore the ROS levels gradually reduced with the state transfering reactions like that of catalase. Unlike the dinuclear Mn(II) complexes which reacted with H_2_O_2_ resulting active Mn^III^Mn^IV^ oxidants, the complex **2** only has one Mn^II^ nuclear, the continuous oxidation of Mn^II^ by H_2_O_2_ may yield as Mn-salen^36^. Ultimately, the levels of ROS would be up-regulated though H_2_O_2_ had been decomposed. Therefore, long-term incubation of **1** scavenged intracellular oxidative stress whereas **2** lead to the accumulation of endogenous ROS for its atypical catalase activity. Zhou *et al.* also reported that the dinuclear Mn(II) complexes of N-substituted di(2-pyridyl-methyl)amine induced cellular ROS by interacting with mitochondria^22^. Mn(II) complex that sharing the same ligand with **2**, also interacts with mitochondria by decreasing the membrane potential^21^.

**Figure 7:**
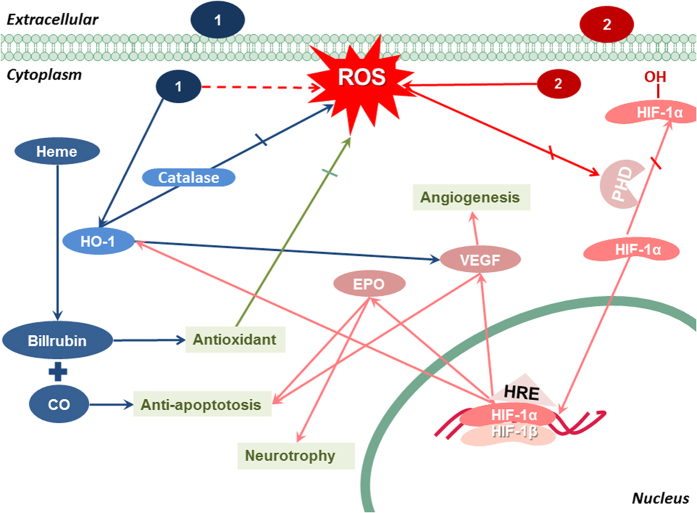
Possible neuroprotection-related signaling pathways of Mn complexes 1 and 2. Relatively short-term pre-incubation of complex **1** and **2** stabilized HIF-1α against degradation through ROS, thereby stimulates HIF and the neurotrophic downstream pathways VEGF, EPO and HO-1. Long-term pre-treatment with **1** eliminates intracellular ROS as catalase does and up-regulates HO-1 expression, which activates anti-apoptotic pathways (including VEGF pathway) by promoting heme cleavage to antioxidant (CO and biliverdin). In comparison, the intracellular ROS generation continuously induced by complex **2** causes cell cytotoxicity.

Not all consequences of ROS production will be deleterious. Our investigations have proved that low dose of H_2_O_2_ was protective for neuronal cells. We also have proved that short-term incubation of **1** and long-term incubation of **2** sustain the intracellular ROS levels. The two compounds both showed HIF-dependent neuroprotective effects for 12-h pre-conditioning. The results are consistent with previous studies in which Ravati *et al.* found that cultured neurons became less sensitive to subsequent insults after short stimulation with ROS generators^37^ and Chang *et al.* pointed out that low dose of H_2_O_2_ is protective against oxidative cell death by activating HIF-1α protein expression, thereby inducing neuroprotective effects of hypoxic/ischemic preconditioning^12^,^38^. In addition, inducing HIF downstream genes enhances metabolism and promotes the growth and repair of neurons^2^. Thus we concluded that short-term pre-treating with **1** and **2** prevented H_2_O_2_-induced neuronal cell apoptosis by conditional ROS stimulation. Low dose of ROS generation triggered the activation of HIF-1α and up-regulation of the anti-apoptotic proteins (Bcl-2) and neuroprotective HIF downstream genes (VEGF, HO-1 and EPO) (Possible neuroprotection-related signaling pathways shown in Fig. 7), indicating that complex **1** and **2** could partly mimic the neuroprotective effects of hypoxic preconditioning.

Neuroprotection of **2** exhibited a time-dependent effect in this study. 12 h pre-conditioning protected neuronal cells more effectively than 24 h preconditioning did, although long-term pre-incubation increased the levels of HIF-1α more evidently. Notably, ROS play dual roles in CNS: In addition to the neuroprotective effects of low levels of free radicals, over-accumulation of ROS in cells, which causes oxidative stress, participates in cell apoptosis and neuronal injury^4^,^5^. Therefore, complex **2**, when employed for pre-treatment for a longer time, was cytotoxicity.

Since over-production of ROS results in cell death by damaging cells, a number of traditional antioxidants and ROS scavenging enzymes have been used as neuroprotective agents to treat stroke and acute ischemia, etc^6^,^7^,^39^,^40^. In this study, Mn complex **1**, which mimics the active site of catalase, successfully prevented the accumulation of ROS with the incubation time prolonged. Especially, both neuroprotective doses of **1** and **2** evidently induced HO-1 protein expression. HO-1 is an early gene responding to an array of pathological conditions including, but not limited to, hypoxia and cerebral ischemia^41^. HO-1 cleaves the heme molecule and produces carbon monoxide and biliverdin as an essential antioxidant^35^. As catalase mimetics, the complex **1** is able to catalyze disproportionation of hydrogen peroxide. Gonzalez-Burgos *et al.* found that stimulating the expression of catalase could up-regulate HO-1 protein levels through the Nrf2-HO-1 pathway^42^. Consequently, by mimicking the overexpression of catalase, preconditioning of **1** stimulated HO-1 expression^43^. Studies indicated that some drug may induce the expression of VEGF gene in the glioma cells probably through the stimulation of HO-1 expression and H_2_O_2_-induced VEGF synthesis was partially dependent on HO-1^44^,^45^. Moreover, HO-1 seems to involve in regulation of angiogenesis^45^, which may explain the inconsistence of VEGF and HIF-1α protein levels. In one word, HO-1 plays an important role in the ROS-HIF-related Mn complexes neuroprotection.

The dual roles that complex **1** stabilizes the protein expression levels of HIF and scavenges the intracellular ROS ensure the compound are both neuroprotective and low cytotoxicity. Thus to design and selection of dinuclear [Mn^III^Mn^IV^(μ-O)_2_μ-OAc] complexes for neuroprotection study are of great practical significance and therapeutic potential.

## Conclusion

In summary, our study provides a dialectic view of ROS on neuroprotection by using two Mn complexes **1** and **2**. Both complexes are capable of regulating intracellular ROS and activate HIF-related signal pathway, but manipulate cellular oxidative stress differently. We have proved that the up-regulation of endogenous ROS in a certain range may enhances cellular antioxidant defenses by stimulating ROS-HIF related pathways, which partly mimics the hypoxic preconditioning effects. Nevertheless, since pre-treatment with **2** for a longer time caused cytotoxicity, Mn complex **1** preconditioning is more effective and appropriate for neuroprotection therapy.

## Methods

### General

PC12 cells were from the Cell Bank of the Chinese Academy of Sciences. Rat hippocampal neurons and neuronal medium were purchased from Sciencell (Sciencell, CA). RPMI 1640 Medium, penicillin, streptomycin and fetal bovine serum (FBS) were from Gibco. NGF-2.5S was from Life Technologies. Trypsin, sodium pyruvate and 3-(4, 5-dimethylthiazol-2-yl)-2,5-diphenyltetrazolium bromide (MTT) were purchased from Sigma. Reactive Oxygen Species Assay Kit was purchased from Beyotime Institute of Biotechnology. VEGF ELISA Kit was bought from Boster (Wuhan, China). The double staining apoptosis Kit was from KeyGEN (Nanjing, China). All other reagents were of analytical grade and all solutions were prepared using Milli-Q deionized water and filtered through a 0.22 micron filter. Complex **1** was synthesized in our group as reported (see Supplementary Fig. S8 online for ESI-MS spectrum)^15^. Complex **2**, provided by Prof. Qiuyun Chen in Jiangsu University, was synthesized as previously reported by her group^21^. Complexes **1** and **2** were dissolved in Milli-Q deionized water as stock solutions, and diluted by culture medium to indicated concentrations.

### Cell culture

Rat hippocampal neurons were thawed in a 37 °C bath and resuspended in poly-L-lysine-coated 35 mm plastic culture dishes with neuronal medium at a final density of 1 × 10^6 ^cells/mL, and kept in a humidified incubator with 95% air +5% CO_2_ at 37 °C for 24 h. Then, cytosine arabinoside (4 μg/mL, final concentration) was added to each dish after 5 days to rid the cultures of dividing glial cells. Later, the culture solution was exchanged twice a week and the cultures were used for experiments after two weeks.

PC12 cells were cultured in RPMI 1640 medium with 10% heat-inactivated FBS, 100 units/ml penicillin, 100 units/ml streptomycin, 1.5 g/L NaHCO_3_, 2.5 g/L glucose and 0.11 g/L sodium pyruvate in a humidified 5% CO_2_ environment at 37 °C. At 70–80% confluence, the cells were harvested after being treated with 0.25% (w/v) trypsin, and then reseeded for expansion. To obtain neuron-like PC12 cells, the cells were maintained with differentiating medium (RPMI 1640 containing 10% heat-inactivated FBS and 25 ng/ml NGF-2.5S). The cells were fed with NGF every other day with differentiating medium until ready for experiment. The cell morphology was observed by a fluorescence microscope (Olympus).

### Cell proliferation assay

MTT assays were applied to test the cell viability. PC12 cells were induced to differentiation by NGF in 96-well plates for 24 h or 36 h, and then the medium was renewed to that contained various concentrations of compound or H_2_O_2_, and incubated for indicated time intervals.

H_2_O_2_ induced cell apoptosis analysis: PC12 cells were induced to differentiation by NGF in 96-well plates for 24 h, and then the medium was renewed to that contained various concentrations of **1** and **2**. After pre-treatment with complexes for 12 h or 24 h, the cells were treated with 100 μM H_2_O_2_ and incubated for 12 h.

To determine cell viability, 20 μl MTT (final concentration: 0.5 mg/ml) was added to each well. After incubation for 4 h, 150 μl of DMSO was added to the cultures to dissolve the crystals. The absorbance at 570 nm was recorded by an automatic enzyme-linked immunosorbent assay plate reader (Thermo Scientific Varioskan Flash).

### Determination of intracellular ROS

Changes in intracellular ROS levels were determined by measuring the oxidative conversion of cell permeable 2′,7′-DCF diacetate (DCFH-DA, Beyotime Institute of Biotechnology) to fluorescent DCF (Ex 488 nm and Em 525 nm). The fluorescence of DCF was measured by a laser scanning microscope or a flow cytometer.

Analysis of DCF fluorescence in collected cells. The cells were harvested by trypsinization, washed three times with PBS, and treated with serum-free culture medium (RPMI 1640) containing 10 μM DCFH-DA. After incubation for 20 min at 37 °C, the cells were washed twice with PBS and measured on a FACScan flow cytometer (BD). ROS levels are expressed as a histogram of the fluorescence generated by 10,000 cells. Mean of the fluorescence was calculated using statistic tool of FlowJo software.

### Western blot analysis

Neuronal cells (1 × 10^5^/ml) were seeded on 60 mm culture dishes in differentiation medium for 24 h, and then the medium was renewed to that contained indicated concentrations of compounds. The cells pre-incubated with drugs for 12 h or 24 h were subsequently treated with 100 μM H_2_O_2_ for 12 h. The extracts of total cellular protein were obtained at 4 °C in lysis buffer containing 20 mM Tris–HCl (pH 8.0), 250 mM NaCl, 0.4 mM Na_3_VO_4_, 1% SDS and 1 × Complete mini protease inhibitor cocktail tablets. Samples were separated by 12% SDS-PAGE and transferred to an immobilon-P transfer membrane (Millipore, USA). Membranes were blocked with 5% nonfat milk in TBS containing 0.1% Tween-20 at room temperature for 1 h, and incubated with primary antibodies (See Supplementary materials online for details of the primary antibodies). The antibodies were diluted in TBS with 5% non-fat milk overnight at 4 °C. Then the blots were incubated with an HRP-conjugated anti-rabbit secondary (1:4000) antibody and an anti-mouse secondary (1:4000) antibody for 1 h (room temperature), respectively. Enhanced chemiluminescence (ECL, Millipore) was performed afterwards.

### ELISA assays

PC12 cells (4 × 10^4^ cells) were seeded on 6-well plates in differentiation medium for 24 h, and then the medium was renewed to that contained indicated concentrations of compounds. The cells pre-incubated with drugs for 12 h or 24 h were subsequently treated with 100 μM H_2_O_2_ for 12 h. Culture supernatants were collected and stored at −80 °C until measurement. The levels of VEGF protein from PC12 cells were detected by an ELISA kit. The detection was performed following the recommendations of the manufacturer by using an automatic enzyme-linked immunosorbent assay plate reader (Thermo Scientific Varioskan Flash).

### RNA isolation and reverse transcription PCR

Total RNA was extracted from tissue samples or cultured cells using Trizol Reagent (Invitrogen) according to the manufacturer’s instructions. The RNA concentration was quantified to two micrograms of each sample through Nanodrop ND-1000 (Thermo Scientific, USA). Each RNA sample was reversely transcribed into cDNA by PrimeScript reverse transcriptase using a primeScript RT-PCR kit. PCR proceeded using the cDNA as a template and TakaRa Taq^TM^ kit by following the manufacturer’s instructions. The number of PCR cycles determined from the plot was 30 for HIF-1α, EPO, HO-1, VEGF and GLUT-3 and 25 for β-actin. The amount of amplified product was detected by 0.1% agarose gel electrophoresis, scanned and analyzed using Quantity One (Bio-Rad, Hercules, CA, USA). The primers are shown in Table S1. Each sample was assayed in triplicate.

### Apoptosis assay

Cells (1 × 10^6^) were collected, washed and resuspended in phosphate buffered saline (PBS), annexin V-fluorescein isothiocyanate (FITC; 5 ml/ml) and propidium iodide (PI) were added and incubated for 20 min at 37 °C. Cells were analyzed by FACScan flow cytometer (Becton Dickinson) with FlowJo software (Tree Star Inc.) and Fluorescence microscope (X-Cite® 120PC Q).

### Plasmid DNA Transfection

Lipofectamine™ 2000 (Invitrogen, Carlsbad, California, America) was used to introduce plasmid DNA into cells. For transfection with the plasmid DNA, vetro cells were seeded into 6-well plates (4 × 10^5^ cells/well) and incubated at 37 °C overnight. When the cultures reached 90% confluence, the plasmid DNA (The Hif-1α siRNA duplex targeted nucleotides of the Hif-1α mRNA sequence (NM001530) and comprised of: sense 5′-CUGAUGACCAGCAACUUGAdTdT-3′ and antisense 5′-UCAAGUUGCUGGUCAUCAGdTdT-3′. The inverted Hif-1α control duplex did not target any gene and comprised of sense 5′-AGUUCAACGACCAGUAGUCdTdT-3′and antisense 5′-GACUACUGGUCGUUGAdTdT-3′. The vector was pSIREN-RetroQ-ZsGreen) was mixed with Lipofectamine™ 2000 and added to the culture medium. After 6 h, cells were washed with phosphate-buffered saline (PBS) and normal medium. Then the cells pre-incubated with drugs for 12 h or 24 h were subsequently treated with 100 μM H_2_O_2_ for 12 h. Cells were analyzed by FACScan flow cytometer (Becton Dickinson) with FlowJo software (Tree Star Inc.) and down regulation effect of plasmid DNA on PC12 cells gene was evaluated by western blot. These did not target any known genes were used as negative controls.

### Statistical Analysis

Data were expressed as mean ± standard deviation (SD). Student’s T. Test was used to compare two independent groups. For comparison between multiple groups (especially for western blot, RT-PCR), were performed for a One-way ANOVA. A value of *p < 0.05, **p < 0.01 was considered to be statistically significant. Statistical analysis was performed with Excel 2010.

## Additional Information

**How to cite this article**: Chen, C. *et al.* Neuroprotection by Polynitrogen Manganese Complexes: Regulation of Reactive Oxygen Species-Related Pathways. *Sci. Rep.* **6**, 20853; doi: 10.1038/srep20853 (2016).

## Supplementary Material

Supplementary Information

## Figures and Tables

**Figure 1:**
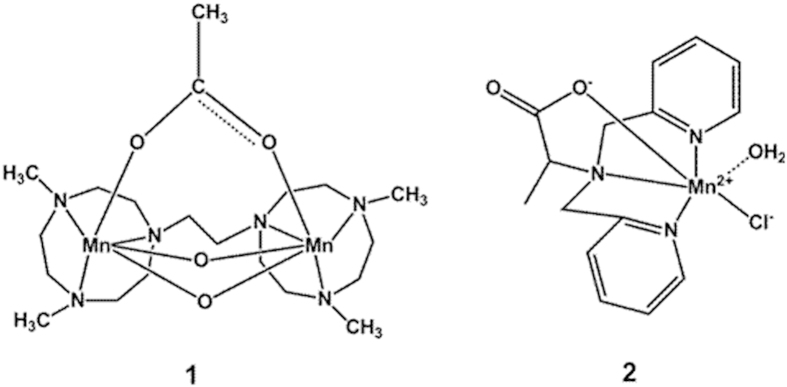
Structural formulas of polynitrogen Mn complexes **1** and **2**.
