# Supplementary material for: Neuroprotection by Polynitrogen Manganese Complexes: Regulation of Reactive Oxygen Species-Related Pathways
Source: Sci Rep. 2016 Feb 9;6:20853. doi: 10.1038/srep20853 (PMC4746657; doi:10.1038/srep20853)
Supplement: Supplementary Information [file srep20853-s1.doc]

**Supplementary information**

Neuroprotection by Polynitrogen Manganese Complexes: Regulation of Reactive Oxygen Species-Related Pathways

Chunxia Chen^a^, Jing Cao^a^, Xiaoyan Ma^a^, Xiaobo Wang^a^, Qiuyun Chen^b^, Shihai Yan^c^, Ningwei Zhao* ^c d^, Zhirong Geng* ^a^ and Zhilin Wang*^a^

^a^ State Key Laboratory of Coordination Chemistry, School of Chemistry and Chemical Engineering, Collaborative Innovation Center of Advanced Microstructures, Nanjing University, Nanjing 210093, P.R. China. Tel.: +86-25-83686082; Fax: +86-25-83317761; E-mail: [wangzl@nju.edu.cn](mailto:wangzl@nju.edu.cn), [gengzr@nju.edu.cn](mailto:gengzr@nju.edu.cn)

^b^ School of Chemistry and Chemical Engineering, Jiangsu University, Zhenjiang 212013, P.R. China

^c^ Department of Pharmacology, Jiangsu Province Hospital of Chinese Medicine, Nanjing 210029, P.R. China. Tel: +86-25-86890278; Fax: +86-25-86555797; E-mail: [sshznw@shimadzu.com.cn](mailto:sshznw@shimadzu.com.cn)

^d^ Biomedical Research Laboratory, Shimadzu (China) Co., Ltd, Shanghai 200052, P.R. China

**Supplementary Materials**

**Antibodies**

The primary antibodies directed to β-Actin[^1^](#_ENREF_1) (1:1000, Beyotime, No. AA128), HIF-1α[^2^](#_ENREF_2) (1:1000, BD Transduction Laboratories^TM^), VEGF^3^ (1:800, LSBio), EPO^4^ (1:100, Santa Cruz Biotechnology), and HO-1^5^ (1:200, Bioss), GAP-43^6^(1:1000, BD Pharmingen) and βIII-tubulin^7^ (1:1000, CST) were used.

**
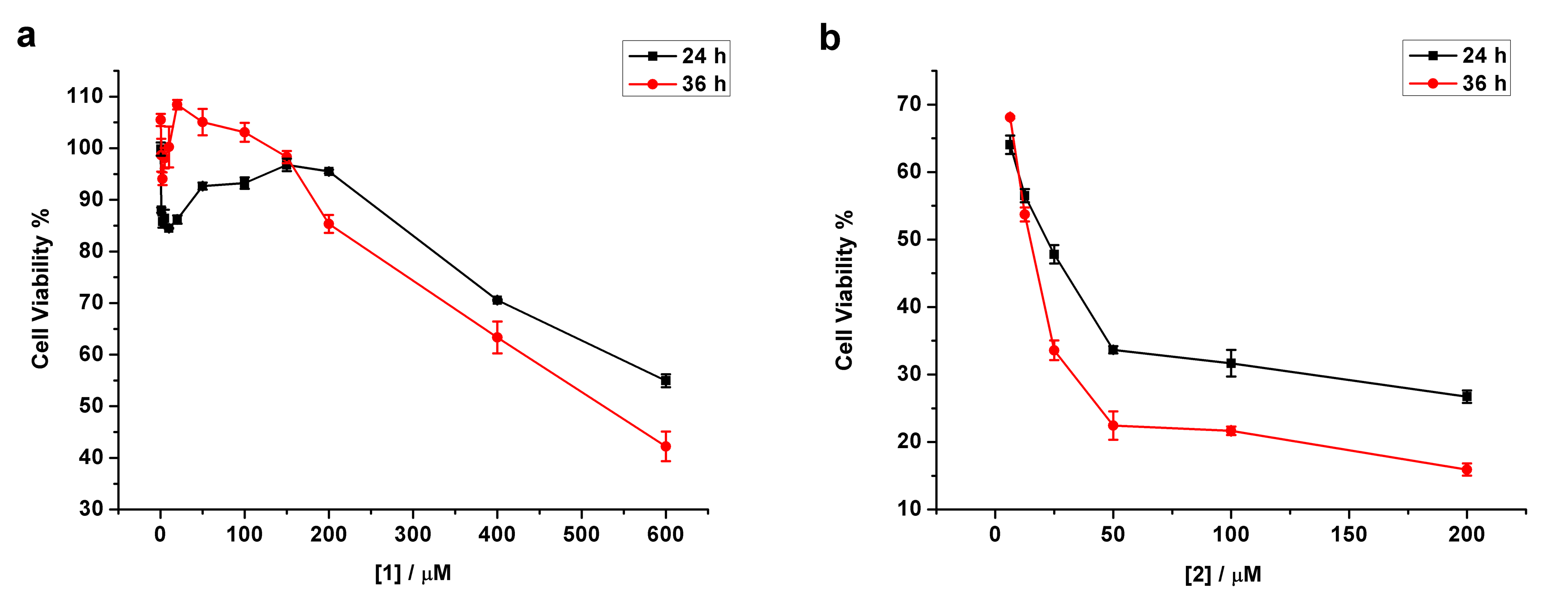
**

**Figure S1** Cytotoxity of **1** and **2** on differentiated PC12 cells. NGF (20 ng/μl) were used to induce PC12 cells differentiation for 24 h, and treated with **1** (a) and **2** (b) for 24 h (black line) and 36 h (red line). Treatment with normal differentiation culture medium without drug stimulation served as control. The cell viability was expressed as the percent (%) of control value by MTT assay. The data are presented as mean ±SD of three independent experiments.


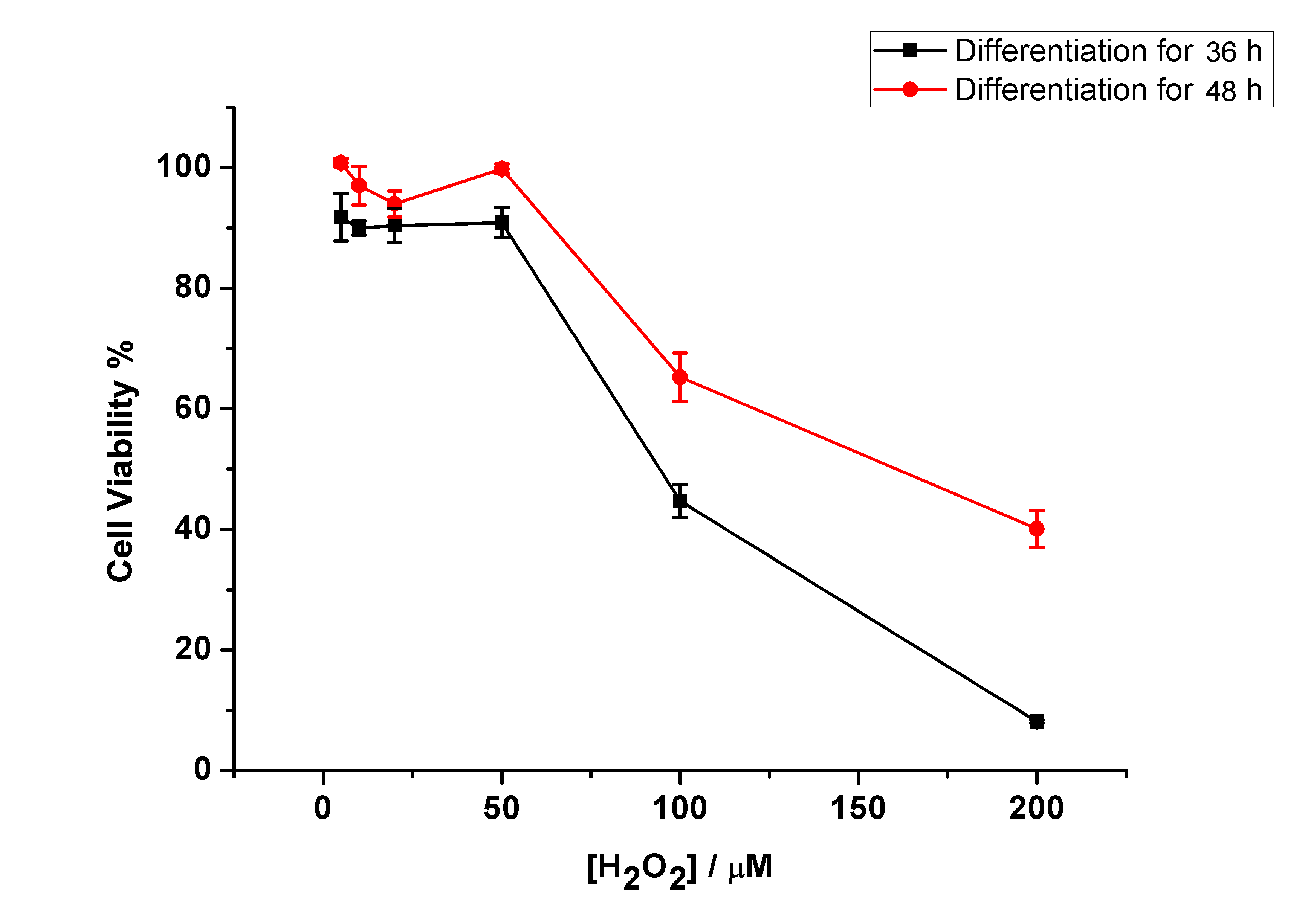


**Figure S2** Effects of H_2_O_2_ concentration on the survival of differentiated PC12 cells. The cells were induced to differentiation for 36 h (red line) or 48 h (black line), and incubated with various doses of H_2_O_2_ for another 12 h. The concentrations of H_2_O_2_ were 6, 10, 20, 50, 100 and 200 μM, respectively. Treatment with normal differentiation culture medium without H_2_O_2_ stimulation served as control. The cell viability was expressed as the percent (%) of control value by MTT assay. The data are presented as mean ±SD of three independent experiments.

**
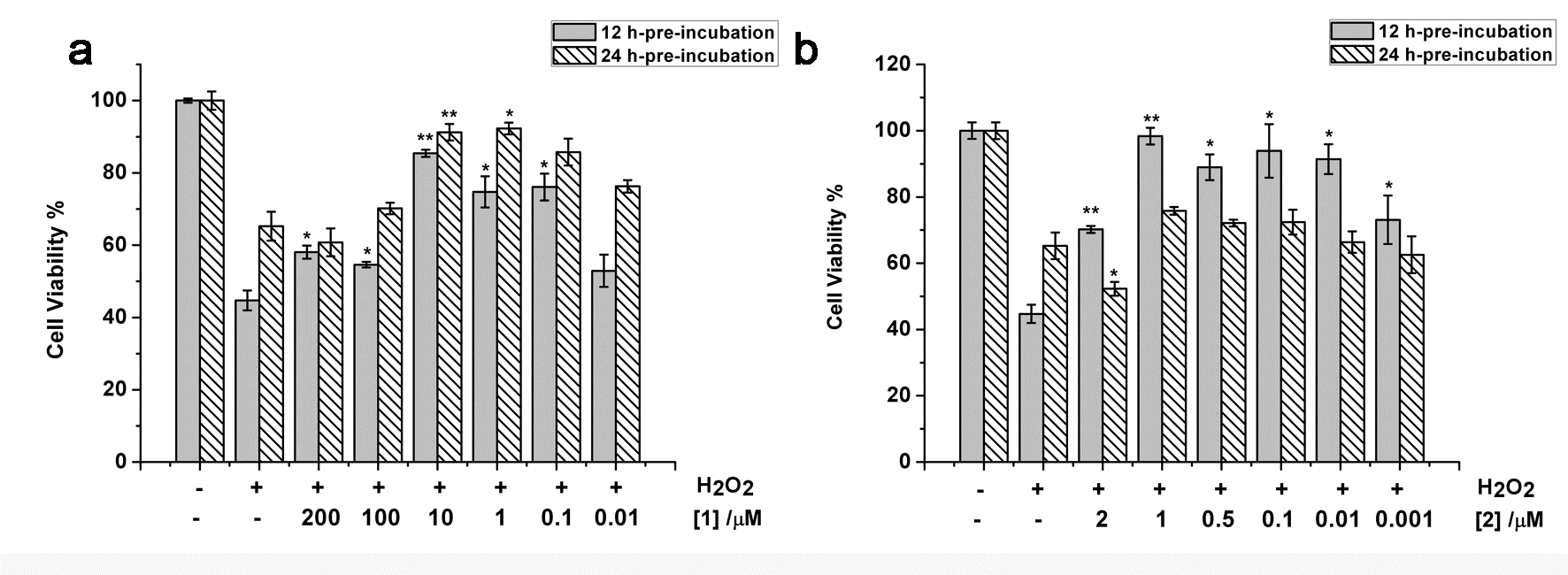
**

**Figure S3** Mn complexes **1** and **2** protected PC12 cells against H_2_O_2_-induced cell apoptosis. The differentiated PC12 cells were pre-incubated with complexes **1** (left) and **2** (right) for 12 h and 24 h, respectively, and treated with 100 μM H_2_O_2_ for 12 h. The pre-incubation concentrations of **1** were 200, 100, 10, 1, 0.1 and 0.01 μM, respectively. The pre-incubation concentrations of **2** were 2, 1, 0.5, 0.1, 0.01, 0.001 μM, respectively. Treatment with normal differentiation culture medium containing neither complexes nor H_2_O_2_ served as control. The cell viability was expressed as the percent (%) of control value by MTT assay. The data are presented as mean ±SD of three independent experiments. (Student’s T. Test, *P < 0.05, **P < 0.01 vs. only H_2_O_2_ stimulation but without any pretreatment)


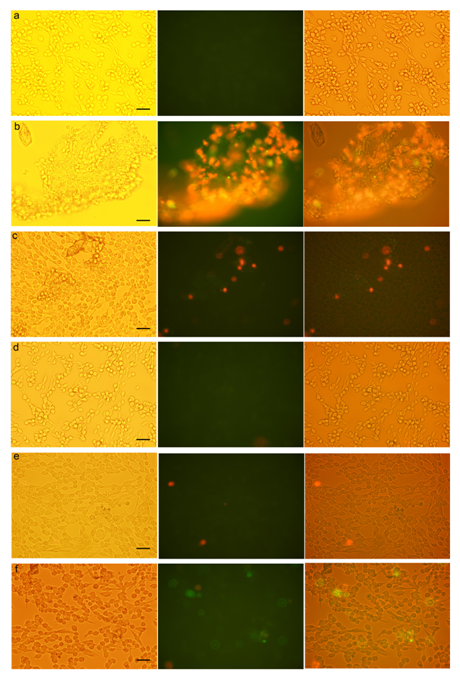


**Figure S4** Mn complexes **1** and **2** protected PC12 cells against H_2_O_2_-induced cell apoptosis. (a) Neuronal medium (control); (b) neuronal medium; (c) **1** (10 μM) for 12 h (d) **1** (10 μM) for 24 h (e) **2** (1 μM) for 12 h (f) **2** (1 μM) for 24 h and then treated with H_2_O_2_ (200 μM) for 12 h (c-f). The scale bar represents 50 μm. The left columns of graphs were under light (×40). The middle columns of graphs were under fluorescence. The right columns of graphs were merged.
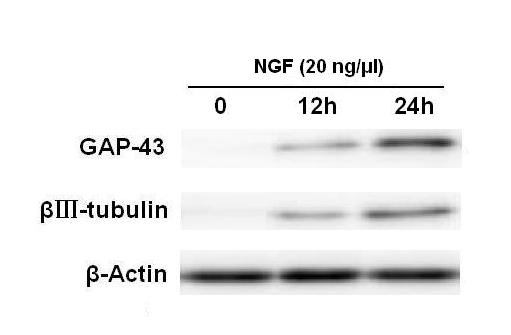


**Figure S5** PC12 cells contain the same neuronal marker with neurons. PC12 was treated with NGF (20 ng /μl) for 0 h, 12 h, 24 h and the neuronal mark: GAP-43 and βIII-tubulin were detected by Western blot.


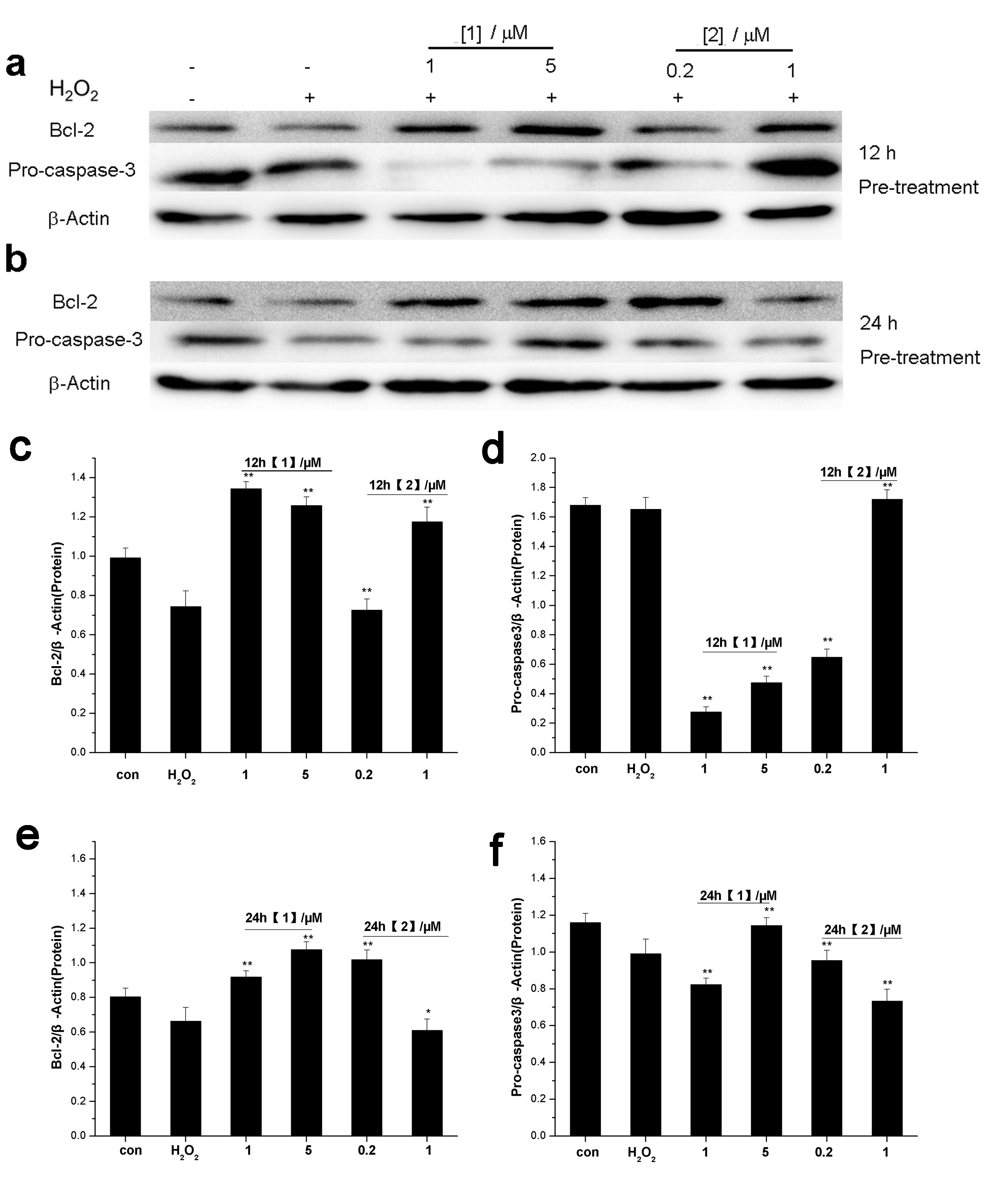


**Figure S6** Preconditioning of Mn complexes **1** and **2** changed Bcl-2 and Pro-caspase-3 protein levels. The differentiated PC12 cells were pre-incubated with **1** and **2** for 12 or 24 h, and treated with H_2_O_2_ (100 μM) for 12 h. The pre-incubation concentrations of **1** were 1 and 5μM, respectively. The pre-incubation concentrations of **2** were 0.2 and 1 μM, respectively. (c) and (e) Statistical analysis of Bcl-2 protein expression levels. (d) and (f) Statistical analysis of Pro-caspase-3 protein expression levels. The expressions of protein levels were given as Bcl-2/β-Actin, Pro-caspase-3/β-Actin ratio. The data are presented as mean ±SD of three independent experiments. (One Way ANOVA, *P < 0.05, **P < 0.01 vs. only H_2_O_2_ stimulation but without any pretreatment**)**


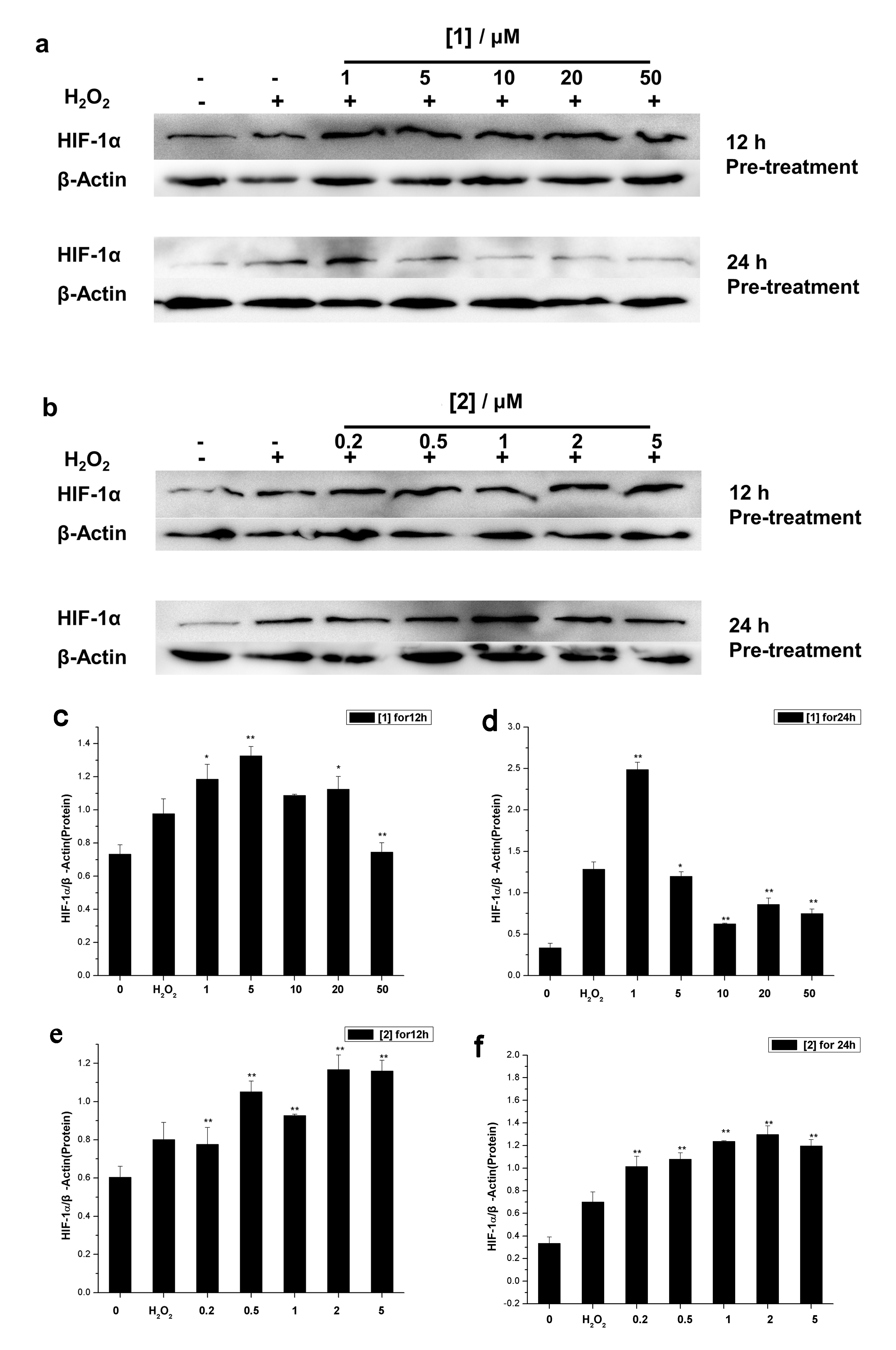


**Figure S7** Preconditioning of Mn complexes **1** and **2** changed HIF-1α levels. The differentiated PC12 cells were pre-incubated with **1** (a) and **2** (b) for 12 or 24 h, and treated with H_2_O_2_ (100 μM) for 12 h. The protein expression of HIF-1α was detected by Western-blot. (c-f) Statistical analysis of HIF-1α gene expression levels and (c) **1** for 12 h (d) **1** for 24 h, (e) **2** for 12 h (f) **2** for 24 h. The pre-incubation concentrations of **1** were 1, 5, 10, 20 and 50 μM, respectively. The pre-incubation concentrations of **2** were 0.2, 0.5, 1, 2 and 5 μM, respectively. Representative immunoblotting of cellular total protein revealed with HIF-1α antibody. β-Actin was used as a loading control.

**
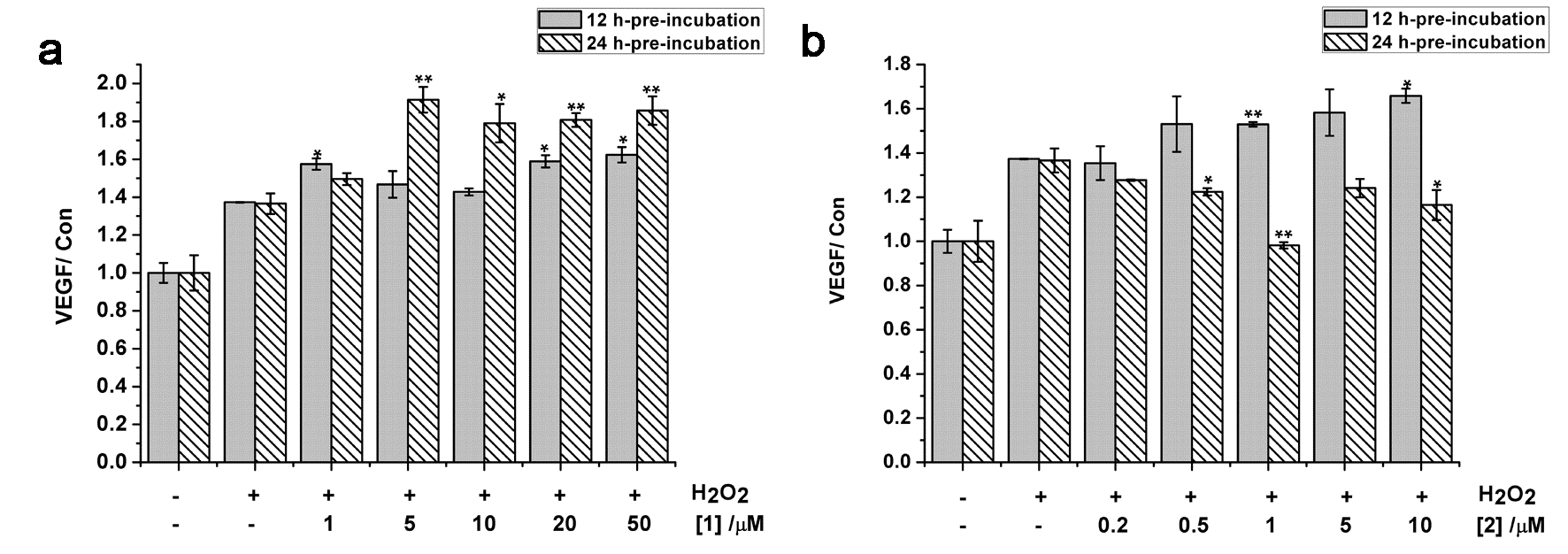
**

**Figure S8** Effects of 1 and 2 pre-conditioning on VEGF secretion. Culture supernatants were collected and the levels of VEGF protein were detected by an ELISA kit. The pre-incubation concentrations of 1 were 1, 5, 10, 20 and 50 μM, respectively. The pre-incubation concentrations of 2 were 0.2, 0.5, 1, 2 and 5 μM, respectively. Treatment with normal differentiation culture medium containing neither complexes nor H_2_O_2_ served as control. The VEGF levels were expressed as the ratio versus control value from the ELISA assay. The data are presented as mean ±SD of three independent experiments. (Student’s T. Test, *P < 0.05, **P < 0.01 vs. only H_2_O_2_ stimulation but without any pretreatment**)**


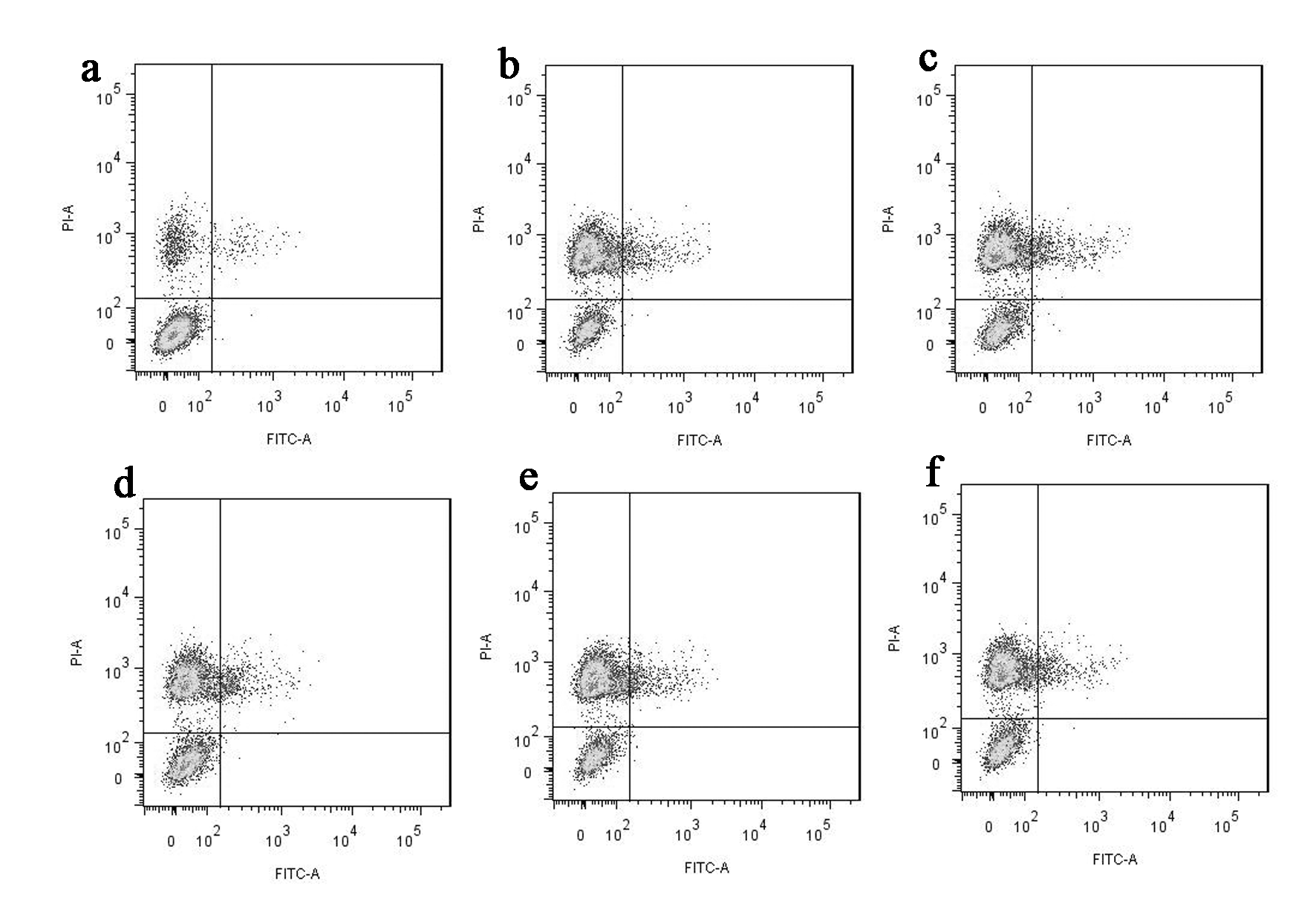


**Figure S9** HIF-1α knockdown induced apoptotic cell death under preconditioning with Mn complexes of neuronal cells. Cell apoptosis detected by a flow cytometer. After 6 h transfection, the cells were pre-incubated with (a) neuronal medium (control), (b) neuronal medium, (c) **1** (10 μM) for 12 h (d) **1** (10 μM) for 24 h, (e) **2** (1 μM) for 12 h and (f) **2** (1 μM) for 24 h and then treated with H_2_O_2_ (200 μM) for 12 h (b-f).


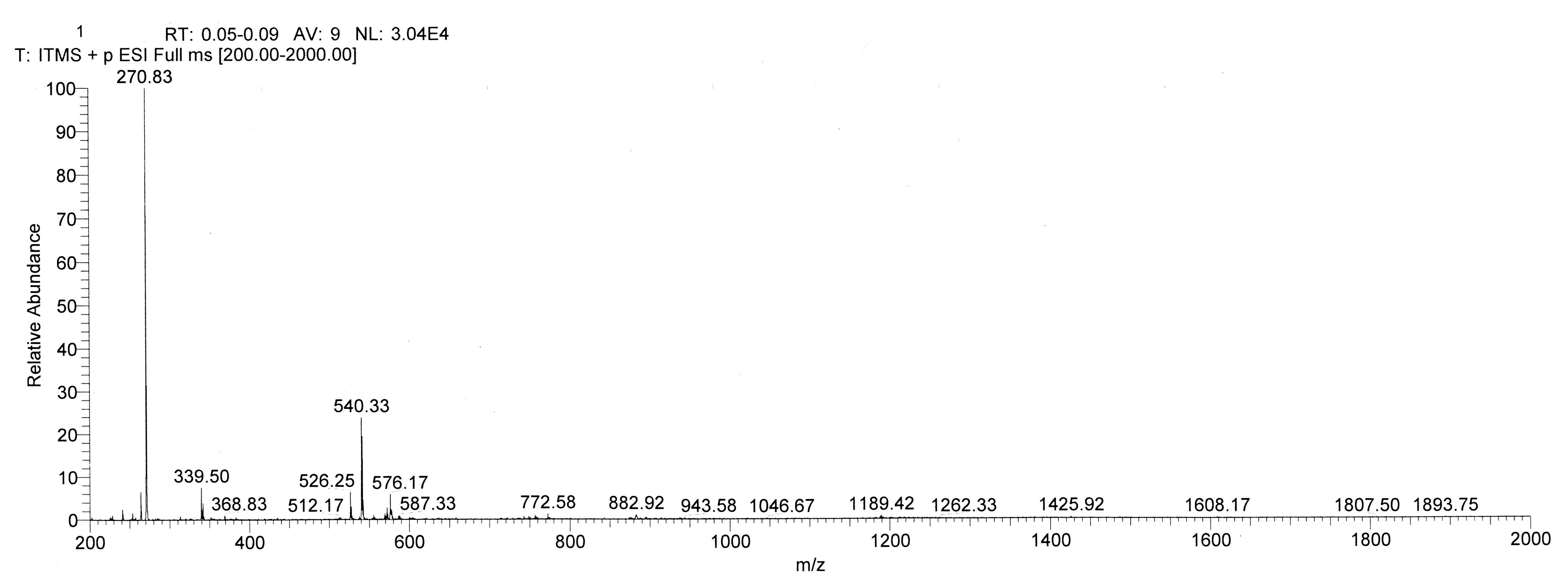


**Figure S10** ESI-MS spectrum of synthesized complex **1**.


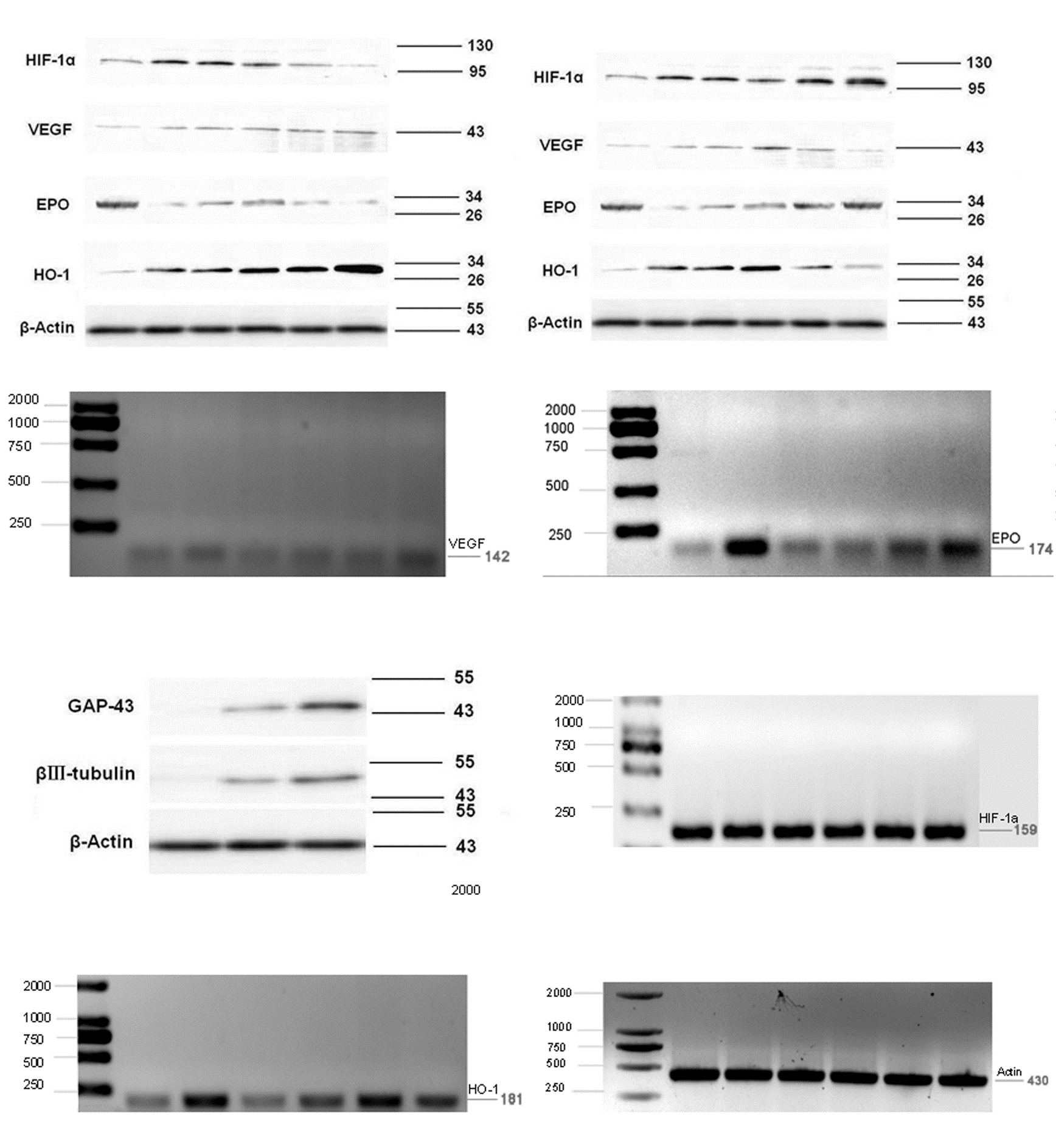


**Figure S11** Full length blots of the cropped HIF-1α, VEGF, β-Actin, EPO, HO-1, GAP-43, βIII-tubulin presented in the main text.

| Name |  | Primer sequence |
| --- | --- | --- |
| HIF-1a | sense | 5' AGTGTACCCTAACTAGCCG 3' |
|  | antisense | 5' CACAAATCAGCACCAAGC 3' |
| VEGF | sense | 5' GAAGGAGGAGGGCAGAAT 3' |
|  | antisense | 5' CACAGGATGGCTTGAAGAT 3' |
| EPO | sense | 5' AATGAAGGTGGAAGAACAGG 3' |
|  | antisense | 5' ACCCGAAGCAGTGAAGTGA 3' |
| HO-1 | sense | 5' CTTTGAGGAGTTGCAGGAGC 3' |
|  | antisense | 5' TGTAAGGACCCATCGGAGAA 3' |
| Actin | sense | 5' GACCTGACTGACTACCTC 3' |
|  | antisense | 5' TCTTCATTGTGCTGGGTGC 3' |

**Table S1**. Primer sequences for the apoptosis factors in reverse transcription PC

**References**

1 Liu, L. *et al.* Notch Signaling Molecules Activate TGF-β in Rat Mesangial Cells under High Glucose Conditions. *Journal of Diabetes Research* **2013**, 1-8 (2013).

2 Arsham, A. M., Plas, D. R., Thompson, C. B. & Simon, M. C. Phosphatidylinositol 3-kinase/Akt signaling is neither required for hypoxic stabilization of HIF-1 alpha nor sufficient for HIF-1-dependent target gene transcription. *J. Biol. Chem.* **277**, 15162-15170 (2002).

3 Jazwaa et al. Effect of heme and heme oxygenase-1 on vascular endothelial growth factor synthesis and angiogenic potency of human keratinocytes. *Free Radical Biology and Medicine.* **40**, 1250–1263 (2006).

4 El Hasnaoui-Saadani, R. *et al.* Epo deficiency alters cardiac adaptation to chronic hypoxia. *Respir. Physiol. Neurobiol.* **186**, 146-154 (2013).

5 Wei, B. et al*.* Regulation of antioxidant system, lipids and fatty acid beta-oxidation contributes to the cardioprotective effect of sodium tanshinone IIA sulphonate in isoproterenol-induced myocardial infarction in rats. *Atherosclerosis* **230**, 148-156 (2013).

6 Chapman ER, Estep RP, Storm DR. Palmitylation of neuromodulin (GAP-43) is not required for phosphorylation by protein kinase C. *J Biol Chem.* **267**,25233-25238(1992).

7 Tischfield MA. et al. Human TUBB3 mutations perturb microtubule dynamics, kinesin interactions, and axon guidance. *Cell* **140**,74-87(2010).
